# Psychometric properties of the Questionnaire for Secondary Traumatization

**DOI:** 10.3402/ejpt.v5.21875

**Published:** 2014-01-09

**Authors:** Katharina Weitkamp, Judith K. Daniels, Fionna Klasen

**Affiliations:** 1 Department of Child and Adolescent Psychiatry, Psychotherapy, and Psychosomatics, University Medical Centre Hamburg-Eppendorf, Hamburg, Germany; 2 Department of Psychiatry, Universitätsmedizin Charité, Berlin, Germany; 3 Department of Psychosomatic Medicine and Psychotherapy, Otto-von-Guericke-University, Magdeburg, Germany

**Keywords:** Secondary traumatization, psychometric properties, questionnaire, compassion fatigue, burnout, vicarious traumatization

## Abstract

**Background:**

During the past several years, there has been a growing interest in the negative effects that providing therapy may have on therapists. Of special interest is a phenomenon called secondary traumatization, which can arise while working with traumatized clients. To develop a simple screening tool for secondary traumatization, a quantitative assessment instrument was constructed using a data-driven approach based on qualitative interviews with affected trauma therapists as well as experienced supervisors in trauma therapy.

**Objective:**

The aim of the current study was to analyze the psychometric properties of the newly developed Questionnaire for Secondary Traumatization (FST) acute and lifetime version and to determine the most appropriate scoring procedure.

**Method:**

To this end, three independent samples of psychotherapists (*n*=371), trauma therapists in training (*n*=80), and refugee counselors (*n*=197) filled out an online questionnaire battery. Data structure was analyzed using factor analyses, cluster analyses, and reliability analyses.

**Results:**

Factor analyses yielded a six-factor structure for both the acute and the lifetime version with only a small number of items loading on differing factors. Cluster analyses suggested a single scale structure of the questionnaire. The FST total score showed good internal consistencies across all three samples, while internal consistency of the six extracted factors was mixed.

**Conclusion:**

With the FST, a reliable screening instrument for acute and lifetime secondary traumatization is now available which is free of charge and yields a sum score for quick evaluation. The six-factor structure needs to be verified with confirmatory factor analyses.

During the past several years, there has been a growing interest in the effects of work-related exposure to graphic details of traumatic events such as in trauma therapists, counselors, and child protection workers. Such vicarious, work-related trauma exposure can result in the emergence of a syndrome called secondary traumatization. In contrast to the construct of burnout, secondary traumatization is thought to arise specifically from the exposure to detailed accounts of traumatic events. Such exposure can be verbal as is the case in trauma therapists or written through graphic descriptions of traumatic incidents as in the case of child protection workers.

Secondary traumatization comprises a set of typical trauma-related symptoms seen in trauma survivors with posttraumatic stress disorder including avoidance, negative mood and cognitions, hyperarousal and intrusions. The concept of secondary traumatization is thus narrower and more specific than the earlier concept of vicarious traumatization, which hinges on conceptual alterations of underlying schemata and has produced highly inconsistent empirical findings (for reviews see Kadambi & Ennis, 2004; Sabin-Farrell & Turpin, 2003).

Several researchers have put forward assessment instruments modeled on assessment instruments for primary trauma victims or based solely on theoretical accounts. Charles Figley co-authored the *Compassion Satisfaction and Fatigue Test* (Stamm & Figley, 1996) and later on the Secondary Traumatic Stress Scales (Bride, Robinson, Yegidis, & Figley, 2004). The former has since been renamed to Professional Quality of Life Scale and comprises three scales, only one of which assesses secondary traumatization. Earlier versions of this test showed collinearity between the three scales. To remedy this problem, the test is currently available in its fifth revision with psychometric evaluation underway. The test by Bride and colleagues (2004) assesses PTSD-like symptoms in a single scale. While test construction was based on psychometrics, further evaluations of the validity of the scale are lacking. Aiming to assess transference symptoms in the general population, Motta and colleagues published the Secondary Trauma Questionnaire (Motta, Hafeez, Sciancalepore, & Diaz, 2001; Motta, Kefer, Hertz, & Hafeez, 1999). This test was not constructed for the assessment of secondary traumatization in professionals specifically and has only been validated on samples from the general population (Motta, Newman, Lombardo, & Silverman, 2004). In summary, to date no reliable and valid assessment instrument based on data rather than theoretical accounts is available, which is designed to specifically assess secondary traumatization in professionals. As a result of this, two core questions still remain unanswered: how many professional mental healthcare providers are affected by secondary traumatization at a given time (point prevalence) and which percentage of mental healthcare providers will develop secondary traumatization at least once over the course of their career (lifetime prevalence). To answer these two questions, an assessment instrument is needed which can be employed using two different time frames as reference points, that is, a point measurement of manifest symptomatology at the time of assessment as well as a *post-hoc* description of the strongest symptomatology ever experienced. While accounting for the obvious limitations of *post-hoc* assessments, only the combination of these two approaches will allow an estimation of the number of therapists affected.

To develop a simple screening tool for both acute and lifetime secondary traumatization in professionals, a quantitative assessment instrument was constructed using a data-driven approach based on qualitative interviews with affected trauma therapists as well as experienced supervisors in trauma therapy (Daniels, 2008). To render this instrument suitable as a screening tool, which could be used routinely in inter- and supervision, it needed to be easily administrable and exercisable within 5 min. For these reasons, the *Questionnaire for Secondary Traumatization* (FST) was modeled on the widely used *Impact of Event Scale* (IES-R; Weiss & Marmar, 1995) in terms of test procedure and scaling. Test development and item selection has previously been described in detail (Daniels, 2006). The aim of the current study was to evaluate the psychometric properties of both the lifetime and the acute versions of the FST. To this end, factor structure and item clustering was studied in one large, independent sample for the lifetime version and in two independent samples for the state version. The rationale for this sample selection was to cover a wider range of professions as well as different levels of exposure to graphic trauma details.

## Methods

### Procedure

Data were acquired in three independent samples. Samples 1 and 3 were used to test the acute version of the FST, assessing symptom severity within the preceding week, while sample 2 was employed to analyze the factor structure of the lifetime version of the FST.

In all three samples, data collection was carried out anonymously employing the online tool UniPark (QuestBack AG, Hürth, Germany). Participants were informed about the proceedings of the study and consented by clicking a continuation button. The online battery consisted of socio-demographic and work-related variables as well as the FST. On average, it took 15 min to fill out the online questionnaire battery. For sample 1, address lists were researched via search engines to attain e-mail addresses of psychotherapists in both private practices and in- and out-patient treatment facilities in Germany. In addition, potential participants were contacted via the e-mail lists of professional associations. A reminder was sent both 6 and 12 weeks after the first contact. Due to this procedure, no data on the participation rate are available. For sample 2, which was administered the lifetime version of the FST, address lists were researched via search engines to attain e-mail addresses of specialized refugee counseling facilities. Additionally, the refugee councils of the German federal states were contacted to obtain their registers of institutions working with refugees. Hence, 748 facilities with an unknown number of employees were contacted via e-mail. In the e-mail, the receivers were informed about the study and the link to the online questionnaire was attached. A precondition for participation was that participants worked with refugees and migrants such as in counseling, therapy, or translation services. For sample 3, participants of a specialized trauma treatment training were contacted by the training institute and requested to partake in the online survey.

### Participants

Three different samples were used to establish the psychometric properties of the instrument. Due to the legal situation in Germany, professionals of different educational backgrounds can work as counselors and psychotherapists. The educational backgrounds of all participants are described below, while basic sample characteristics are given in Table 2.

#### Sample 1—counselors and psychotherapists

Sample 1 consisted of 371 counselors and psychotherapists. Almost all participants (*n*=365, 98.4%) had a degree in higher education: 169 psychologists, 72 social educators, 37 psychiatrists, 36 other medical doctors, 36 psychotherapists, 26 pedagogues, 15 registered alternative psychotherapists (Heilpraktiker), 5 social workers, as well as some ergo- and dance therapists, political scientists, or certified supervisors (each group less than 5). The participants without a university degree consisted of five nurses and one occupational therapist. A total of 88.7% of this sample (*n*=329) reported having undergone specific psychotherapy training, while 66.0% (*n*=245) reported further trauma-specific education, with 31.3% (*n*=116) having attended more than one trauma-focused education. The most prevalent trauma-specific approach was Eye Movement Desensitization and Reprocessing (EMDR, *n*=139, 37.5%) besides a number of other approaches, for example, psychoimaginative trauma therapy or structural dissociation. Therapists gave on average 18.4 therapy sessions per week. They reported that on average 51.0% of their clientele were traumatized and that 49.2% were traumatized under the age of 18 years. At the time of assessment, 50.4% of their current clients were in treatment due to trauma-related disorders. Nearly 74.9% reported receiving some form of regular supervision.

#### Sample 2—refugee counselors

Sample 2 consisted of the staff of several refugee counseling centers across Germany (Schmidt, 2012). They provided assistance and counseling on topics, such as health, housing, and education. The sample consisted of 196 counselors of which 186 (94.9%) had a degree in higher education: 87 social educators, 47 social workers, 21 pedagogues, 19 psychologists, as well as some medical doctors, psychotherapists, business economists, and political scientists (each group less than 5). The group without a university degree (*n*=9) consisted of three nurses, three clerks, two students, and one foreign language clerk. In this sample, 19.4% (*n*=38) had partaken in specific psychotherapy education and 15.8% (*n*=31) reported having undergone further trauma-specific education. Eighty-six of the participants (43.9%) worked full-time in refugee counseling with an overall average of 31.1 hours per week (SD = 9.61).

#### Sample 3—psychotherapists in training for trauma therapy

Sample 3 consisted of 80 therapists currently undergoing trauma therapy training. All but one participant had a degree in higher education: 46 psychologists, seven psychiatrists, 12 other medical doctors, seven social educators, eight psychotherapists, as well as some pedagogues, registered alternative psychotherapists (Heilpraktiker), and pastors (each group less than 5). All participants reported being trained in psychotherapy and 63.8% (*n*=51) reported further trauma-specific education in addition to the one they were receiving in during recruitment. They provided on average 19.9 therapy sessions per week. On average, 26.4% of their current clientele were traumatized and 26.0% had been victimized under the age of 18 years. 66.3% reported receiving some form of regular supervision.

Across all three samples, the majority of the participants was female (80.8%) and on average 47.5 years of age (SD = 9.45) with 15.9 years of work experience (SD = 9.34). On average, 44.2% of their clients were traumatized. 55.6% reported receiving some form of regular supervision.

### Measurements

The *Questionnaire for Secondary Traumatization* comprises 31 items consisting of questions regarding symptoms of the four PTSD symptom clusters according to the DSM-5, as well as items covering sense of threat and safety behavior (see Table 1). Two versions of this questionnaire are available – a lifetime version retrospectively assessing the week with the highest level of distress across the career and a state version assessing symptom severity within the previous week. These two versions only differ regarding the instruction, while the items are identical.

**Table 1:** FST items

			*M*		
			
	Item	Scale	Sample 1	Sample 2	Sample 3
1.	I ruminated on what happened to the client.	General	2.60	3.39	2.30
2.	I unwillfully thought about what happened to the client.	Intrusion	2.28	2.96	2.08
3.	I had intrusive images or sensations that are connected to what I was told.	Intrusion	1.77	2.38	1.74
4.	I felt like reliving my clients’ experience.	Intrusion	1.32	1.67	1.45
5.	I was afraid something bad could happen to me.	Feeling of threat	1.41	1.45	1.41
6.	I had disturbing dreams that are connected to what I was told.	Intrusion sleep	1.30	1.65	1.24
7.	I witnessed in my dream what happened to my client.	Intrusion sleep	1.09	1.27	1.08
8.	I dreamt of what happened to my client as if it were happening to me.	Intrusion sleep	1.04	1.13	1.02
9.	When I was reminded of my clients’ experience I felt distressed.	Intrusion	2.25	2.39	1.86
10.	When I was reminded of my clients’ experience I reacted with physiological arousal and stress.	Intrusion	1.94	1.99	1.49
11.	I tried not to think of my clients’ experience.	Active avoidance	1.89	2.23	1.45
12.	I avoided objects, places or activities that reminded me of my clients’ experience.	Active avoidance	1.22	1.34	1.01
13.	I felt alienated from other people.	Neg cog and mood	1.63	1.52	1.27
14.	I withdrew from other people or was less active than normally.	Neg cog and mood	1.91	1.59	1.55
15.	My emotions were less intense than normally.	Neg cog and mood	1.62	1.51	1.44
16.	I was less interested in activities that I normally enjoy a lot.	Neg cog and mood	1.72	1.65	1.49
17.	I focused more on my personal safety.	Feeling of threat	1.78	1.64	1.65
18.	I took additional precautions for my personal safety.	Feeling of threat	1.46	1.35	1.25
19.	I felt threatened or followed.	Feeling of threat	1.25	1.21	1.11
20.	I had trouble falling asleep or woke up more often than I do normally.	Hyperarousal	1.92	2.00	1.76
21.	I was jumpy.	Feeling of threat	1.46	1.43	1.36
22.	I had trouble concentrating.	Hyperarousal	1.83	1.81	1.53
23.	I was on edge.	Hyperarousal	2.07	2.06	1.84
24.	We had conflicts and arguments in my team.	Hyperarousal	1.79	1.90	1.51
25.	I was less interested in sex or enjoyed it less.	Hyperarousal	1.95	1.78	1.74
26.	Due to my job stress I drank more alcohol or took more drugs.	General	1.45	1.39	1.40
27.	I was afflicted by thoughts or visual imaginations of assaults against me or people I love.	Feeling of threat	1.39	1.40	1.23
28.	My health was impaired, i.e., by headaches, nausea, infections.	Hyperarousal	1.91	1.75	1.68
29.	I recalled or dreamt of my own trauma history more often than normally.	Intrusions sleep	1.69	1.53	1.59
30.	I experienced myself as being depressed.	Neg cog and mood	1.80	1.66	1.58
31.	I thought about suicide.	Neg cog and mood	1.09	1.11	1.01

Items are rated on a Likert scale from 1 = *never* to 5= *very often* (adopting the scaling of the IES-R). In the lifetime version, participants are instructed to specify the period in their career, during which they experienced the highest level of distress. They are then prompted to retrospectively rate how often the 31 symptoms occurred in the worst week during this specified time period. In contrast, the acute version of the FST simply prompts participants to rate the occurrence of these 31 symptoms over the course of the last week. In the first step, the psychometric properties of the lifetime version of the FST were evaluated in a retrospective epidemiological study of the individual's most severe symptom level over the course of their whole career. A total of 1,124 therapists gave accounts of both their experiences with trauma work-related stress as well as primary trauma exposure and associated symptoms (Daniels, 2006). The analysis of the FST item characteristics yielded medium to high discriminatory power levels for all items with sufficient variance in item difficulty as well as a high internal consistency (*α*=0.94). Both the cluster analysis and the factor analysis suggested computing one sum score instead of sub-scales best represents the data. The cluster analysis also gave indications of preliminary diagnostic criteria: participants scoring between 65 and 82 points were classified as experiencing moderate secondary traumatization, while participants scoring above 82 were classified as suffering from severe secondary traumatization (Daniels, 2006). Applying these diagnostic criteria, 29.1% of the sample was retrospectively diagnosed as having suffered from moderate or severe secondary traumatization at one time throughout their career.

In addition to the FST, the participants were asked socio-demographic questions regarding their age, gender, country of residence, education, psychotherapy and trauma-specific training, current employment and position, working hours, supervision, current and overall percentage of traumatized clients, and percentage of clients traumatized under the age of 18 years.

### Analyses

Data of the three different samples were analyzed separately. Since data collection was carried out online and progression to the next webpage was dependent on completion, there were no missing data regarding the FST in the included data sets. Reliability was established with an analysis of the internal consistency using Cronbach's *α* for the total score, as well as the later established factors from the factor analysis. Additionally, bivariate inter-item correlations were calculated.

Exploratory factor analysis with the principal axis method and Varimax rotation was utilized to examine the underlying structure of the newly developed FST more closely. Factor analyses were carried out with the two large samples, sample 1 and sample 2. Eigenvalues > 1 and factor loadings >0.60 were considered as meaningful, and loadings >0.40 as sufficient (Bortz, 2005). Factor agreement and meaningful interpretability of the emerging factors across the samples were set as additional criteria to compare the two samples. If an item showed high loadings on similar factors across the two samples, the item was allocated to this factor. If an item showed differing loadings between the two samples these were noted in the results section. The internal consistence of the newly established factors was than tested with the remaining sample, sample 3.

Cluster analyses were carried out to complement the data structure analysis. Agglomerative hierarchical cluster analyses were employed in each sample separately to form content clusters. Individual FST items were grouped into clusters using single linkage (nearest neighbor) method and Euclidean distances. The agglomeration stages are visually displayed as dendrograms. Cluster formations were plotted along a scaled between-stage distance axis from 0 = individual items to 100 = unitary cluster of all symptoms. Visual inspection of the dendrogram was used to determine the appropriate formations of clusters using the following rules: Items should be grouped into a cluster: (1) if their dendrograms converged within a 10-unit window from the previous merger on the cluster-distance axis; and (2) if the convergence occurred before unit 50 (Kircanski, Woods, Chang, Ricketts, & Piacentini, 2010). If no differentiation into separate clusters is achieved applying these criteria, a very strict, high-resolution criterion is used for further exploration: as a sudden increase in the agglomeration level indicates an increase in complexity, (3) the most homogenous items will be identified by setting the cut-off directly before the largest distance in the dendrogram (Wiedenbeck & Züll, 2001). To provide evidence for the robustness of the clusters, cluster analyses were carried out with the three subsamples independently to determine whether similar cluster subgroups were found in each of the subgroups. All analyses were conducted using SPSS 18.0.

## Results

The mean FST total score was 51.80 (SD = 14.89) across samples (see Table 2). Total FST score was significantly lower in sample 3 (psychotherapists) than in samples 1 and 2 (1–3: *p*≤0.007; 2–3: *p*<0.001; see Table 1). Sample 2 reported the highest FST total scores. As mentioned above, sample 2 was the sample with the instruction to report on the period of their career with the highest level of distress. Each of the three samples differed significantly in terms of age (1–2: *p*<0.001; 1–3: *p*<0.001; 2–3: *p*<0.001), duration of work experience (1–2: *p*<0.001; 1–3: *p*≤0.001; 2–3: *p*<0.001), and average percentage of traumatized clients (1–2: *p*<0.001; 1–3: *p*<0.001; 2–3: *p*≤0.013). Sample 2 (refugee counselors) consisted of significantly less females than sample 1 (counselors and psychotherapists) (*z*=2.042; *p*≤0.020). Furthermore, sample 3 (psychotherapists) had significantly less currently traumatized clients and less clients with an age of traumatization under 18 years compared with sample 1 (psychotherapists and counselors) (*F*=36.680; *p*<0.001). The refugee counselors reported less psychotherapy training (1–2: *z*=21.206; *p*<0.001; 2–3: *z*=28.536; *p*<0.001), less trauma-specific training (1–2: *z*=14.012; *p*<0.001; 2–3: *z*=8.039; *p*<0.001), and less ongoing supervision (1–2: *z*=4.592; *p*<0.001). Overall, the refugee counseling sample (sample 2) was on average younger with less work experience, and reported the lowest rate of traumatized clients compared with the other two samples (see Table 2). The refugee counseling (sample 2) received significantly less supervision than the counselors and psychotherapists (sample 1).

**Table 2:** Characteristics of the three samples of therapists and counselors

	Sample 1 *n*=371	Sample 2 *n*=196	Sample 3 *n*=80		
			
	Counselors and psychotherapists	Refugee counselors	Psychotherapists		
			
	Mean (SD)	Mean (SD)	Mean (SD)	Sig. test	*Post-hoc*
Age (years)	48.33 (SD = 8.81)	43.91 (SD = 10.15)	52.53 (SD = 7.33)	*F*=28.973 *p*<0.001	1–2**, 2–3**, 1–3**
Gender (female)	309 (83.3%)	150 (76.5%)	64 (80.0%)	χ^2^ =3.821 *p*≤0.148	1–2*
Country					
Germany	357 (96.2%)	196 (100%)	70 (87.5%)	χ^2^ =33.211 *p*<0.001	
Austria	7 (1.8%)	–	2 (2.5%)		
Switzerland	6 (1.6%)	–	8 (10.0%)		
Others	1 (0.3%)	–	–		
Work experience (years)	17.19 (SD = 9.52)	11.39 (SD = 7.48)	21.20 (SD = 8.23)	*F*=44.107 *p*<0.001	1–2**, 2–3**, 1–3**
Psychotherapy training (%)	88.7	19.4	100	χ^2^=303.138 *p*<0.001	1–2**, 2–3**
Trauma training (%)	66.0	15.8	63.8	χ^2^=135.772 *p*<0.001	1–2**, 2–3**
Average percentage of traumatized clients (%)	50.97 (SD = 29.25)	39.03 (SD = 29.86)	27.84 (SD = 19.03)	*F*=26.988 *p*<0.001	1–2**, 2–3*, 1–3**
Current percentage of traumatized clients (%)	50.38 (SD = 31.69)	n/a	26.43 (SD = 21.671)	*F*=41.474 *p*<0.001	1–3*
Percentage of clients victimized <18 years (%)	49.24 (SD = 32.76)	n/a	25.95 (SD = 22.47)	*F*=36.680 *p*<0.001	1–3*
Supervision (%)	74.9	55.6	66.3	χ^2^=22.131 *p*<0.001	1–2*
FST total score	51.08 (14.95)	54.14 (15.42)	46.10 (11.49)	*F*=8.484 *p*≤0.001	2–3**, 1–3**

* Note: *p* <.05

** *p* <.001.

### Sample 1

*Inter-item correlations* of the FST were mostly moderate (see Supplementary file). The lowest bivariate correlation was *r*=0.03 between item FST7 and FST26. Highest correlations were between FST14 and FST16 (*r*=0.72**).

The *Factor analysis* yielded the following results for sample 1. Six factors were extracted which explained a total of 59.2% of variance (see Table 3). The first factor (eigenvalue 5.359) represented mainly items covering negative cognitions and mood as well as hyperarousal. The second factor (eigenvalue 3.736) included items covering mainly intrusion and active avoidance. The third factor (eigenvalue 3.515) included items covering feelings of threat. The fourth factor (eigenvalue 2.433) included all items on intrusion symptoms during sleep together with an item on avoiding places and activities. The fifth factor (eigenvalue 1.842) covered sleeping problems, alcohol consumption, and suicidal ideation. Factor six (eigenvalue 1.469) consisted of only one item measuring re-experiencing. Items FST3 and FST20 showed double-loadings >0.40. Item FST29 had factor loadings <0.40 on all factors, the highest loading was 0.359 on factor 1.

**Table 3:** FST rotated factor loadings – sample 1 counselors and psychotherapists. Highest factor loading per item in bold

	Component					
	
Item	1 Neg cog and mood/hyperar	2 Intrusions/active avoidance	3 Feelings of threat	4 Intrusions sleep/active avoidance	5	6
1.	0.103	**0.671**	0.166	0.065	0.085	0.004
2.	0.107	**0.714**	0.150	0.167	0.115	0.182
3.	0.161	**0.503**	0.233	0.206	0.107	0.461
4.	0.135	0.259	0.171	0.133	0.095	**0.666**
5.	0.088	0.227	**0.650**	0.114	−0.010	0.120
6.	0.183	0.290	0.194	**0.569**	0.109	0.022
7.	0.163	0.044	0.102	**0.792**	−0.047	0.084
8.	0.014	0.117	0.087	**0.756**	0.022	0.055
9.	0.316	**0.711**	0.139	0.168	0.067	0.091
10.	0.286	**0.664**	0.146	0.126	0.120	0.197
11.	0.196	**0.707**	0.172	0.100	0.006	−0.018
12.	0.195	0.220	0.258	**0.469**	0.116	0.120
13.	**0.659**	0.114	0.159	0.234	0.154	0.326
14.	**0.800**	0.067	0.191	0.075	0.014	0.229
15.	**0.769**	0.136	0.109	0.042	0.045	0.150
16.	**0.803**	0.185	0.215	0.070	0.058	0.145
17.	0.241	0.158	**0.767**	0.108	0.083	−0.025
18.	0.154	0.104	**0.797**	0.107	0.202	−0.046
19.	0.167	0.131	**0.737**	0.169	0.080	0.082
20.	**0.455**	0.223	0.180	0.181	**0.456**	−0.097
21.	0.364	0.160	**0.456**	0.065	0.335	0.098
22.	**0.644**	0.177	0.188	0.219	0.318	−0.047
23.	**0.640**	0.281	0.228	0.120	0.196	−0.213
24.	**0.402**	0.249	0.249	0.341	0.158	−0.288
25.	**0.645**	0.330	0.100	0.117	0.003	−0.201
26.	0.170	0.233	0.141	−0.065	**0.669**	−0.080
27.	0.219	0.255	**0.513**	0.152	−0.051	0.274
28.	**0.421**	0.376	0.190	0.221	0.263	−0.127
29.	**0.359**	0.144	0.373	0.296	0.145	0.240
30.	**0.631**	0.213	0.148	0.076	0.345	0.117
31.	0.151	−0.065	0.042	0.119	**0.725**	0.351

*Reliability*—internal consistency was high for the FST total score with Cronbach's α = 0.94. Additionally, internal consistency was calculated for the suggested factors from the factor analysis, except for the last factor which consisted only of one item. The first three factors showed high reliability (1: *α*=0.91; 2: *α*=0.84; 3: *α*=0.82), factor 4 and 5 showed lower reliability scores (5: *α*=0.64; 6: *α*=0.51).

*Cluster analysis* was employed to study the underlying structure of the FST more closely. All items were allotted to a single cluster following criterion 2, and all items converged within the 50-unit window on the cluster-distance axis as required for criterion 1 (agglomeration coefficient between 5.916 and 18.628). The largest sudden increase in complexity could be observed after the convergence of the first two items. These first two items to converge were measuring intrusion symptoms (FST7 “I witnessed in my dream what happened to my client” and FST8 “I dreamt, what happened to my client, as if it would be happening to me”). The remaining items converged with small increases in complexity throughout, indicating the homogeneity of these remaining items.

### Sample 2

*Inter-item correlations* of the FST were mostly moderate (see Supplementary file). The lowest bivariate correlation was *r*=0.09 between item FST8 and FST26. Highest correlations were between FST14 and FST16 (*r*=0.76**).

The *Factor analysis* yielded a six-factor solution of the FST which explained a total of 63.6% of variance (see Table 4). Very similar to the first factor in sample 1, this first factor (eigenvalue 5.044) covered items on negative cognition and mood as well as hyperarousal. The second factor (eigenvalue 3.444) included items assessing feelings of threat and is thus almost identical to factor 3 of sample 1. The third factor (eigenvalue 3.399) covered items on inclusion similar to factor 2 of sample 1 on intrusions and active avoidance. The fourth factor (eigenvalue 2.821) included mixed items such as measuring intrusion, hyperarousal, and avoidance. In sample 1, these items are represented by factor 1 and factor 2. The fifth factor (eigenvalue 2.511) comprised items on intrusion symptoms during sleep, which correspond to factor 4 in sample 1. The sixth factor (eigenvalue 2.503) included items on somatic complaints, their own traumatic history, and suicidal ideation. Items FST6, FST9, FST19, FST22, FST26, FST28, and FST29 showed double-loadings > 0.40, indicating the interconnectedness of the extracted factors.

**Table 4:** FST rotated factor loadings – sample 2 refugee counselors, lifetime version. Highest factor loading per item in bold

	Component					
	
Item	1 Neg cog and mood/hyperar	2 Feelings of threat/active avoidance	3 Intrusions	4 Mixed/active avoidance	5 Intrusions sleep	6
1.	0.277	−0.067	**0.749**	0.128	0.117	−0.050
2.	0.173	−0.051	**0.799**	0.099	0.225	0.093
3.	0.039	0.303	**0.773**	0.037	0.022	0.105
4.	−0.017	0.381	**0.610**	0.099	0.006	0.298
5.	−0.049	**0.691**	0.278	0.256	0.142	0.100
6.	0.156	0.194	**0.482**	0.321	**0.428**	0.009
7.	0.156	0.236	0.279	0.030	**0.680**	0.131
8.	0.062	0.243	0.112	0.065	**0.817**	0.076
9.	0.291	0.043	**0.479**	**0.552**	0.160	0.085
10.	0.246	0.278	0.343	**0.566**	0.146	−0.095
11.	0.255	0.250	0.381	**0.501**	−0.049	−0.038
12.	0.292	**0.426**	0.055	0.159	0.281	0.084
13.	**0.624**	0.303	0.134	0.082	0.117	0.126
14.	**0.793**	0.188	0.092	0.240	0.128	0.137
15.	**0.706**	0.312	0.171	0.219	0.060	0.030
16.	**0.778**	0.125	0.184	0.242	0.088	0.229
17.	0.155	**0.666**	0.125	0.254	0.238	0.154
18.	0.348	**0.666**	−0.067	0.136	0.183	0.165
19.	0.330	**0.621**	−0.016	0.081	0.407	−0.032
20.	**0.552**	0.011	0.261	0.277	0.343	0.175
21.	**0.472**	**0.443**	0.049	0.152	0.375	0.316
22.	**0.602**	0.108	0.132	0.406	0.294	0.224
23.	0.399	0.202	0.095	**0.649**	0.148	0.158
24.	0.175	0.159	−0.040	**0.628**	0.018	0.373
25.	**0.639**	0.142	0.116	0.255	0.073	0.364
26.	**0.456**	0.135	0.121	−0.015	−0.088	**0.456**
27.	0.357	**0.583**	0.271	−0.072	−0.014	0.115
28.	0.350	0.033	0.070	**0.441**	0.081	**0.499**
29.	0.211	0.231	0.004	0.147	**0.483**	**0.525**
30.	**0.445**	0.127	0.069	0.285	0.243	**0.623**
31.	0.141	0.109	0.120	0.026	0.096	**0.764**

*Reliability*—internal consistency was very high for the FST total score with a Cronbach's *α*=0.94. Additionally, internal consistency was calculated for the suggested factors from the factor analysis of sample 1, except for the last factor which consisted only of one item. The first three factors showed high reliability (1: *α*=0.92; 2: *α*=0.83; 3: *α*=0.84), factors 4 and 5 showed lower reliability scores (5: *α*=0.70; 6: *α*=0.57). These alphas were comparable to the internal consistency results in sample 1.

Using *cluster analysis*, item convergence of the FST was studied more closely. All items converged within the 50-unit window on the cluster-distance axis (criterion 1; agglomeration coefficient between 6.164 and 14.933), but no separable clusters with a distance of more than 10 points on the scale (criterion 2) emerged. The two largest sudden increases in complexity each covered 4 points on the distance scale. The first was observed after the convergence of the first five items. This cluster comprised the items FST7, FST8, FST18, FST19, and FST31. These items measure the frequency of intrusive reliving, the feeling of being threatened personally and suicidal ideation. After these five items, only small increases in complexity were observed up to the last four items, which again converged with a sudden increase in complexity. These items were FST1, FST2, FST3, and FST11. These items are characterized by their general nature, as they measure rumination and intrusive thoughts about clients as well as intentional thought suppression.

### Sample 3

Sample 3 was utilized to test the reliability of the six-factor structure. We followed the suggested factor structure of sample 1 (acute version), since sample 3 was also assessed with the acute version of the FST.

Inter-item correlations of the FST were mostly low to moderate (see Supplementary file). The lowest bivariate correlation was *r*= − 0.21 between item FST24 and FST31. Highest correlations were between FST7 and FST8 (*r*=0.87**).

*Reliability*—internal consistency of the total FST score was very high in this sample with a Cronbach's *α*=0.92. Reliability analysis of the extracted factors was high for Factors 1–4 (1: *α*=0.89, 2: *α*=0.84; 3: *α*=0.81; 4: *α*=0.73) and low for factor 5 which included only three items (*α*=0.32). Factor 6 consisted of only one item and was thus excluded from the reliability analysis.

*Cluster analysis* revealed that all items converged within the 50-unit window on the cluster-distance axis (criterion 1; agglomeration coefficient between 1.414 and 8.062) and were allotted to only one cluster according to criterion 2. The largest increase in complexity was observed after the convergence of the first four items, which all converged within four units on the distance scale. These items covered intrusive re-experiencing (FST7 “I witnessed in my dream what happened to my client”, FST8 “I dreamt what happened to my client as if it would be happening to me”, FST12 “I avoided objects, places, or activities that reminded me of my clients’ experiences”), as well as suicidal ideation (FST31 “I thought about suicide”). After the convergence of these four items, only small increases in complexity were observed.

## Discussion

The aim of the current study was to evaluate the psychometric properties of the newly developed FST in three independent samples and to determine the best scoring procedure. Overall, the results suggest the scale to be multi-factorial, but to be best represented by a total score summed across all items. This was found to be true for both the lifetime and the state version of the FST.

The samples differed in terms of age (sample means ranging from 44 to 52 years) and, accordingly, work experience (sample means ranging from 11 to 20 years). In addition, the three samples targeted counselors and psychotherapists working with traumatized people under different conditions. In samples 1 and 2, the majority of the participants was trained for psychotherapy and reported having received trauma-specific training, in contrast to sample 3, where this was only true for a minority. General exposure levels to traumatized clients also differed between the samples, with a quarter versus half of the clients being traumatized. Of the traumatized clientele, a quarter versus a half of these patients had experienced the traumatization under the age of 18. We thus successfully recruited diverging samples, allowing us to base the methodological evaluation on professionals with differing levels of exposure, expertise, and work experience. Importantly, the two samples assessed with the acute version of the questionnaire (samples 1 and 3) differed regarding their current exposure levels at the time of assessment (50.4% vs. 26.4%), due to the fact that sample 3 was recruited during specialized training for trauma therapy.

First, internal consistency was very high in all three samples. With regard to the internal consistency for the suggested factors, sample 1 showed high reliability (between *α*=0.82 and *α*=0.91), for the first three factors, while factors 4 and 5 showed lower reliability scores (*α*=0.64 and *α*=0.51, respectively). These results were mirrored in sample 2. Again, the first three factors showed high reliability (between *α*=0.83 and *α*=0.92), while factors 4 and 5 showed lower reliability scores (*α*=0.70 and *α*=0.57, respectively). This phenomenon is partly due to the low number of items loading on these factors. However, future studies should try to replicate these factor structures using confirmatory factor analyses.

Secondly, the results of the acute and the lifetime version yielded six factors, which differed only slightly across the samples. In both samples, most items assessing negative cognition and mood as well as hyperarousal loaded on the first factor. Most intrusion items clustered together with items assessing active avoidance on a single factor (factor 2 in sample 1 and factor 3 in sample 2), while the items assessing intrusive dreams loaded on a separate factor (factor 4 in sample 1 and factor 5 in sample 2). Items assessing feelings of threat also clustered together on a single factor (factor 3 in sample 1 and factor 2 in sample 2). While the sequence of the corresponding factors 2 and 3 measuring feelings of threat and intrusions was reversed between the samples, this might not be seen as problematic, since the eigenvalues were in a comparable range.

The main difference in factorial structure between the samples concerned factor 4 identified in sample 2. Items loading on factor 1 in sample 1 comprise this factor. It remains to be tested by future studies if this is due to the different time frame for reference, that is, if this constitutes a stable difference between the test forms, or is dependent on the specific sample studied in this investigation. Future studies should therefore run confirmatory factor analyses on both versions of the test in the same sample to elucidate this finding.

As the factor loadings did not place all items in the same factor groupings across samples, we presented suggested sub-scale as well as total item scores to provide a basis for further examination of the sub-scales in future research and screening. Rather than proposing two slightly differing sub-scale versions, future investigation could establish differing cut-offs for the acute and the lifetime FST versions. The internal factor structure of sample 1 was mirrored in the internal consistency analysis of sample 3. Cronbach's *α* was on a similar level for factors 1–4. Factor 5, the factor with only three items (sleeping problems, suicidal ideation, and alcohol consumption) showed considerably lower internal consistency in sample 3 (*α*=0.32) than in sample 1 (*α*=0.51). This cross-examination of the internal consistency supports factors 1–4 and suggests a further examination of the sub-scale structure with confirmatory factor analysis, particularly for factor 5.

Third, the computation of a total score across all FST items was supported by the results of the *cluster analyses*. Similar results were found for the two different instruction types, lifetime vs. acute distress. In all three samples, all items converged within the 50-unit window. Thus, no differentiation into separate clusters was indicated. Overall, only small sudden increases in complexity were observed. However, there was one group of items that converged first across the three data sets indicating that these items tended to co-occur with one another: “I witnessed in my dream what happened to my client” (FST7), “I dreamt, what happened to my client, as if it would be happening to me” (FST8), “I avoided objects, places of activities that reminded me of my clients’ experience” (FST12), and “I thought about suicide” (FST31). These are the core items indicating severe secondary traumatization, covering the key symptoms of intrusion and avoidance. If someone endorses these items to the point of suicidal ideation, it is very likely that this person will also show the other symptoms of FST. These items seem to indicate the degree of the secondary traumatization rather than some qualitative aspect of the phenomenon.

Across the three data sets, the last items to converge were the following, though not as consistently as the first items: “I ruminated on what happened to the client” (FST1) and “I unwillfully thought about what happened to the client” (FST2). These items describe relatively general symptoms which are often reported in the absence of any other, more severe, ST symptoms. Similar to the cluster of items converging first, this cluster is an indicator of the severity of secondary traumatization rather than the quality of an aspect of the phenomenon. In addition, the item “We had conflicts and arguments in my team” (FST24) showed late convergence. This could be due to only a subset of each sample actually working in a team. As there was no option in the questionnaire to mark this items as not applicable, participants primarily working on their own would have rated this as occurring very seldom, which could explain the low overall correlation between this items and the remaining symptoms within this group of participants. Unfortunately, we have no way of ascertaining this hypothesis as the amount of teamwork was not assessed in this study.

## Limitations

The current study aimed to evaluate the internal structure and validity of the FST. Since the study relied on the anonymous self-report of the participants, no data on the actual mental health status and the traumatic stress experiences is available. Hence, no conclusions regarding the external validity of the instrument can be drawn at this stage of the development of the FST. Similarly, due to the cross-sectional nature of the three studies, no re-test reliability was computed.

Due to the recruitment through an anonymous online tool, we cannot discern if these numbers adequately reflect the socio-demographic characteristics of the contacted population or if they are influenced by a response bias. For instance, the majority of the participants was female. This could mirror the gender ratio in counseling and psychotherapy or a bias towards female readiness to participate in this kind of survey. However, the fact that the FST was tested in three different populations yielding comparable results across those independent samples indicates the robustness and wide applicability of the FST.

## Conclusions

The newly developed state FST showed good internal consistencies. Cluster analyses and factor analyses suggested a single scale structure of the questionnaire. With the FST, a reliable instrument is available which is free of charge and can be used to screen for secondary traumatization in high-risk populations. Both the lifetime and the acute distress versions seem comparably valid to assess symptoms of secondary traumatization. The FST is therefore suitable for regular utilization in supervisory contexts to identify subjects at risk for the development of chronic symptomatology and enable supervisors to intervene in a timely manner.
